# Transcriptome sequencing, de novo assembly, characterisation of wild accession of blackgram *(Vigna mungo* var*. silvestris)* as a rich resource for development of molecular markers and validation of SNPs by high resolution melting (HRM) analysis

**DOI:** 10.1186/s12870-019-1954-0

**Published:** 2019-08-16

**Authors:** Avi Raizada, J. Souframanien

**Affiliations:** 1 Nuclear Agriculture and Biotechnology Division, BARC, Trombay, Mumbai, Trombay 400085 India; 2 Homi Bhabha National Institute, Training School Complex, Anushakti Nagar, Mumbai, Anushakti Nagar 400094 India

**Keywords:** Wild blackgram, Transcriptome, Simple sequence repeat (SSR), SNP genotyping, High resolution melting (HRM)

## Abstract

**Background:**

Blackgram [*Vigna mungo* (L.) Hepper], is an important legume crop of Asia with limited genomic resources. We report a comprehensive set of genic simple sequence repeat (SSR) and single nucleotide polymorphism (SNPs) markers using Illumina MiSeq sequencing of transcriptome and its application in genetic variation analysis and mapping.

**Results:**

Transcriptome sequencing of immature seeds of wild blackgram, *V. mungo* var*. silvestris* by Illumina MiSeq technology generated 1.9 × 10^7^ reads, which were assembled into 40,178 transcripts (TCS) with an average length of 446 bp covering 2.97 GB of the genome. A total of 38,753 CDS (Coding sequences) were predicted from 40,178 TCS and 28,984 CDS were annotated through BLASTX and mapped to GO and KEGG database resulting in 140 unique pathways. The tri-nucleotides were most abundant (39.9%) followed by di-nucleotide (30.2%). About 60.3 and 37.6% of SSR motifs were present in the coding sequences (CDS) and untranslated regions (UTRs) respectively. Among SNPs, the most abundant substitution type were transitions (Ts) (61%) followed by transversions (Tv) type (39%), with a Ts/Tv ratio of 1.58. A total of 2306 DEGs were identified by RNA Seq between wild and cultivar and validation was done by quantitative reverse transcription polymerase chain reaction. In this study, we genotyped SNPs with a validation rate of 78.87% by High Resolution Melting (HRM) Assay.

**Conclusion:**

In the present study, 1621genic-SSR and 1844 SNP markers were developed from immature seed transcriptome sequence of blackgram and 31 genic-SSR markers were used to study genetic variations among different blackgram accessions. Above developed markers contribute towards enriching available genomic resources for blackgram and aid in breeding programmes.

**Electronic supplementary material:**

The online version of this article (10.1186/s12870-019-1954-0) contains supplementary material, which is available to authorized users.

## Background

Blackgram, *Vigna mungo* (L.) Hepper, popularly known as urdbean or mash, is a grain legume domesticated from *V. mungo* var*. silvestris* [1]. Blackgram is a self-pollinating annual diploid (2*n* = 2*x* = 22) crop with a genome size of approximately 574 Mbp [2]. India is the largest producer and consumer of blackgram and the world’s major contributor in blackgram production. It is grown in 5.44 million hectares of area with an annual production of about 3.56 million tons during 2017–2018 and an average productivity of 655 kg per hectare [3]. It is extensively grown in varied climatic conditions and soil types in India. While substantial yield improvements have been made in this crop, realizing the yield potential is still a major challenge posed by biotic, abiotic stresses and non-availability of suitable plant types for different growing conditions. Genetic improvement, either by traditional or molecular methods, has been hampered by the limited genomic resources coupled with narrow genetic diversity in the elite gene pool. There is a need to accelerate progress of crop improvement programmes with the use of molecular markers in conventional breeding more appropriately molecular breeding.

Molecular marker studies in this crop are restricted to the random markers and SSR markers developed from related crop species. In blackgram, genetic relationships and diversity among germplasm collections have been investigated mostly using random amplified polymorphic DNA (RAPD) [4], inter simple sequence repeat (ISSR) [4], amplified fragment length polymorphism (AFLP) [5, 6] and SSR or microsatellite [6, 7] markers. DNA markers linked to mungbean yellow mosaic virus (MYMV) disease and powdery mildew resistance gene(s) were tagged with ISSR, SSR and RAPD respectively [8–10]. In blackgram, two linkage maps were published till date which were based on population derived from inter-subspecific crosses and developed with 145 (61 SSR, 56 RFLP, 27 AFLP) and 428 (254 AFLP, 47 SSR, 86 RAPD and 41 ISSR) markers respectively [11, 12]. As earlier reported genetic markers have limited use and were less informative, there is a need to develop new molecular marker types. SSRs and SNPs are the markers of choice for molecular genetic study in crops because of their co-dominant, multi-allelic, abundance in genome, easy scoring features and most importantly are PCR-based.

The next-generation sequencing (NGS) technologies lead to development of large-scale genomic resources, including transcriptome sequence data, molecular markers, genetic and physical maps making trait mapping and marker-assisted breeding more feasible [13]. Transcriptome sequencing allows precise expression studies at genomic level for non-model organisms and organisms that lack genome sequence information. Transcriptome sequencing using NGS technologies allow quick and inexpensive SNPs and SSR discovery within the coding regions which are more likely to be related with the various biological functions [14–16]. Simple sequence repeats (genic-SSRs / EST-SSRs) and SNPs derived from transcribed region of genome have been applied for genetic diversity analysis, genetic map construction, association mapping analysis, marker-assisted selection (MAS) breeding and quantitative trait locus mapping in many well-studied species [17, 18]. In addition, EST-SSRs show a higher level of transferability to closely related species than genomic SSR markers [19]. Advances in SNP detection techniques allow high-throughput assays and consequently high-density genome-wide scans [20, 21]. High resolution melting (HRM) analysis has been used to detect SNPs, INDELs and SSRs present in small amplicons [22].

Wild species (crop wild relatives or CWR) represent a large pool of genetic diversity, constitutes an important source of useful gene(s) required in breeding programs. Exploratory studies suggest that about 150 species of *Vigna* exist in nature and 22 *Vigna* species including cultivated and wild are found in Indian gene centre [23]. In the improvement of blackgram, the sub gene pool of wild type consisting of *Vigna mungo* var. *silvestris* can be a valuable source for resistance against biotic and abiotic stresses. MYMV resistant types in *V. mungo* var. *silvestris* accession IW3390 have been reported [24]. Accession INGR10133 of *Vigna mungo* var. *silvestris* have been identified as a potential source for bruchid resistance and found to be under the control of two dominant duplicate genes [25, 26]. Quantitative trait loci (QTLs) associated with bruchid (*Callosobruchus maculatus)* resistance were mapped with the help of inter-subspecific mapping population generated by crossing *V. mungo* var. *mungo* (cv. TU 94–2, bruchid susceptible) and *V. mungo* var. *silvestris* (bruchid resistant) [8, 27].

Transcriptome analysis (TA) of CWR and their cultivated counterparts can be used to determine the difference in the expression patterns of genes involved in the genomic basis of several processes such as adaptation of the CWR in varied environments, crop domestication and diversification, evolutionary process and genomic regions, which lost diversity in domestication bottlenecks [28, 29]. In addition, RNA Seq of developing seeds of elite cultivars reveals molecular basis of yield [30], maturity [31], resistance to diseases, insect-pests [32], abiotic stress tolerance [33], transcription modulation behind seed development and nutrient accumulation [34], that can aid in introgression of these agronomic traits in other cultivars through development of molecular markers. Understanding of gene/genome evolution, genome organization and genetic variations needs transcriptome sequencing of more species [28]. Till date, reported SSR markers for blackgram are from single variety (TU94–2) transcriptome and other related *Vigna* species and no SNPs markers has been reported in blackgram. Transcriptome sequencing of wild species of blackgram has not been reported. In this study, transcriptome sequencing of Trombay Wild (TW) (*Vigna mungo* var. *silvestris*), a wild accession of blackgram was carried out with following objectives: (1) Development of a large expressed sequence dataset from immature seeds of the wild relative *Vigna mungo* var. *silverstri*s using the Illumina paired-end sequencing technology; (2) To analyze the frequency and distribution of SSRs in transcribed region of the wild progenitor (3) To develop a large set of genic-SSR and SNP markers; (4) Studying the intra specific variation revealed by genic-SSR markers and (5) SNPs genotyping through (HRM) analysis and validate through re-sequencing.

## Results

### Illumina paired end sequencing and de novo assembly of transcriptome

The transcriptome sequencing of blackgram wild accession (TW) generated 19,690,124 high-quality reads (base quality more than 20) which accounts for 2,970,339,385 bases (2.97 GB). Illumina reads have been submitted to the NCBI short read archive under the submission ID. SRR 5931432, SRX3091690 and study accession SRP115376. High quality reads were assembled and resulted in 40,178 transcript contigs (TCS). The length of contigs ranged from 280 to 6445 bp with an average length of 446 bp (Additional file 1: Figure S1). There were 6398 (15.9%) TCS with length between 200 to 299 bp and 15,966 (39.7%) with length between 300 to 399 bp. TCS with length more than 500 bp and 1000 bp accounted for 26.6% (10,694 TCS) and 2.2% (905 TCS) respectively.

### Functional annotation

**Fig. 1:**
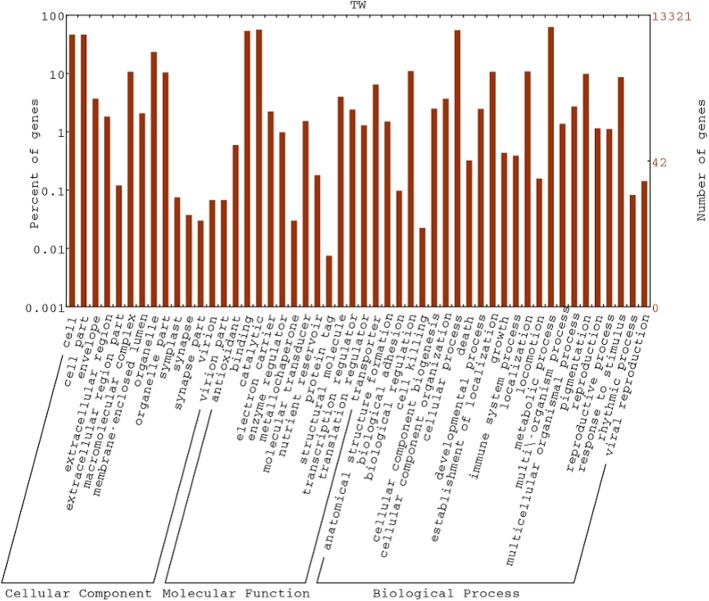
GO Annotation analysis for wild blackgram CDS using BLAST2GO algorithm

A total of 38,753 (96.4%) CDS were predicted from 40,178 TCS with the help of ORF Finder and annotated through sequence similarity search against the NCBI non-redundant (nr) protein database using BLASTx algorithm. Significant similarity to known proteins in nr database were found for 28,984 (74.79%) CDS and 9769 (25.2%) CDS had no hits in the database. Majority of the CDS showed similarity with *Phaseolus vulgaris* (27%) followed by *Citrus clementina* (16%) (Additional file 2: Figure S2). CDS were functionally annotated by Blast2GO for consistent gene annotations and assigned with gene names, gene products, EC numbers and Gene Ontology (GO) numbers. Out of 28,984 CDS, 28,106 CDS were assigned to gene ontology classes with functional terms (Additional file 3: Table S1). Among all the three classes, maximum numbers of CDS were assigned to molecular function (11,544, 41.1%), followed by biological process (10,330, 36.8%) and cellular component (6232, 22.2%) (Additional file 4: Figure S3). Most of the CDS from molecular function class were assigned to binding and catalytic activities. In biological process category, majority of the CDS fall under cellular processes, metabolic processes followed by establishment of localization (Fig. 1). CDS were mapped to a total of 140 unique KEGG pathways. Out of which majority are falling into purine metabolism (524), starch and sucrose metabolism (217), glycolysis/gluconeogenesis (183), pyrimidine metabolism (181) and cysteine and methionine metabolism (160).

### Identification, frequency and distribution of SSRs in blackgram TCS

**Fig. 2:**
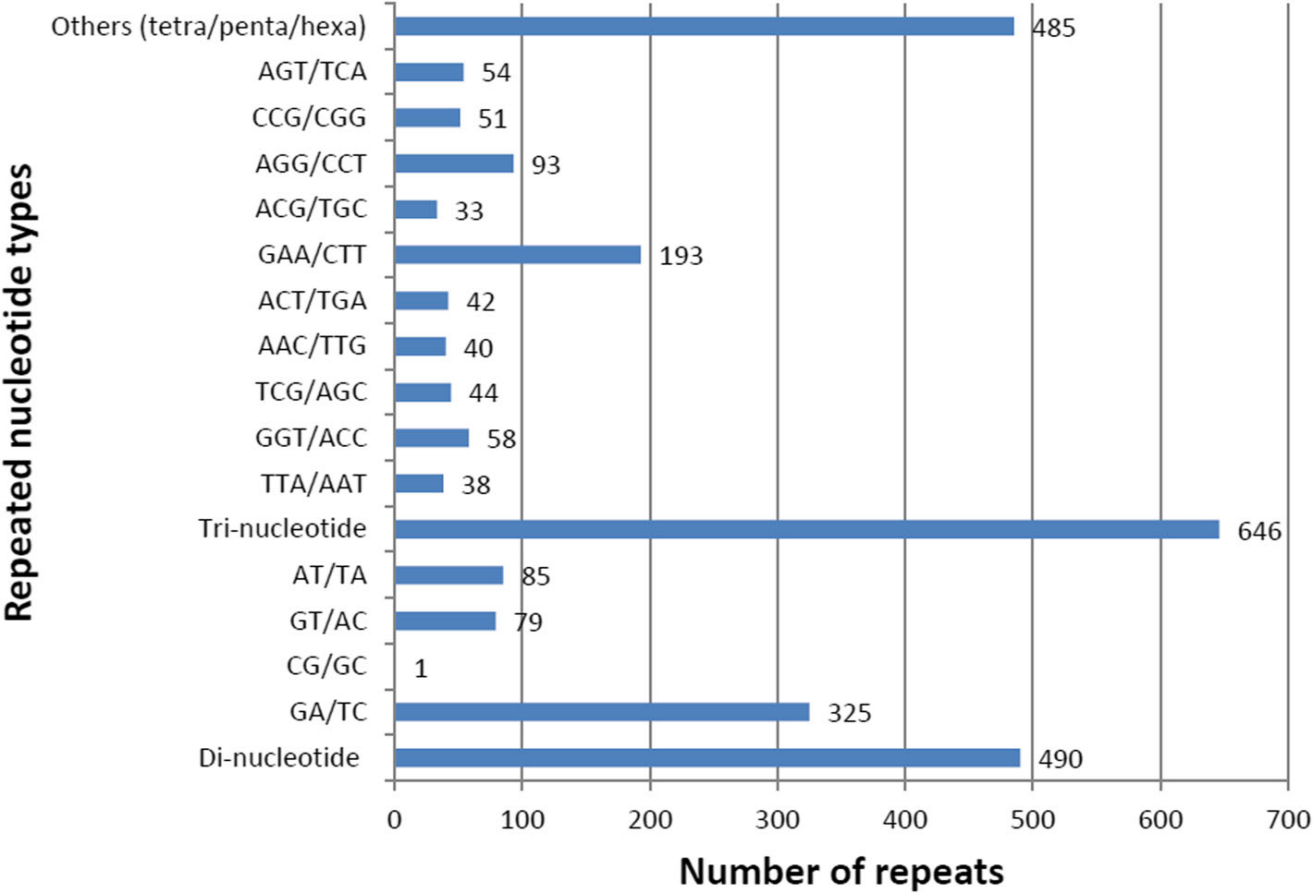
Simple sequence repeat (SSR) types and distribution in the wild blackgram transcriptome

**Fig. 3:**
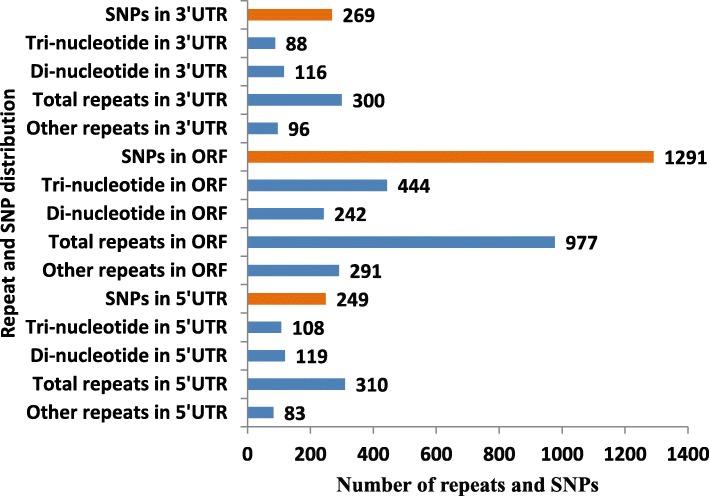
Frequency and distribution of SSRs and SNPs in coding sequence and untranslated region (UTRs) of blackgram TCS

Out of 40,178 TCS, 1621 SSRs were identified in 1339 (3.3%) TCS, with an average frequency of one SSR per 11.1 kb. Tri-nucleotide repeats were found to be most abundant (646,39.9%) followed by di-nucleotide (490,30.2%) and together constitute 70.1% of the identified SSRs (Fig. 2). GAA/CTT nucleotides were found to be the largest SSR motif (29.9%) compared to AGG/CCT (14.4%) and GGT/ACC nucleotides (9%). Repeat number ranged from 6 to 47 for di-nucleotides, 5–52 for tri-nucleotides, 4–18 for tetra-nucleotides, 4–30 for penta-nucleotides and 3–10 for hexa-nucleotides. In di-nucleotide SSR class, GA/TC alone constitute 66.3% followed by AT/TA and GT/AC which accounts for 17.3 and 16.1% respectively, whereas CG/GC occurs only once (1,0.2%) in the transcriptome dataset. Identified SSRs were classified into three classes on the basis of their position in TCS as whether lying in CDS, 5’UTR, or 3’UTR. Sequence analyses revealed 977 (60.3%) SSR repeats were present in the CDS and 610 (37.6%) SSR repeats were found in untranslated regions of which 19.1 and 18.5% were accounted for 5’UTR and 3’UTR respectively (Fig. 3). Both tri-nucleotide (444,68.7%) and di-nucleotide (242,49.4%) repeats were preferentially present in the CDS. Thirty four SSR loci could not be classified into any of the three classes as no ORF was found for their respective transcripts.

### Development of genic-SSR markers

A total of 1621 SSR motif were identified and PCR primer pairs were successfully designed for 1171 SSR loci. Details of primers sequence, expected product size and T_m_ for 1171 genic-SSR primer pairs is provided in Additional file 5: Table S2. Primers could not be designed for 450 SSR loci. One hundred primer pairs were initially screened in six blackgram genotypes (TW, RIL68, Nayagarh, LBG-17, KU96–3, TAU-1) and an amplification rate of 58% was observed. Of these, 31 and 19 SSR primers showed polymorphism and null alleles respectively.

### Genetic diversity analysis in blackgram germplasm

**Fig. 4:**
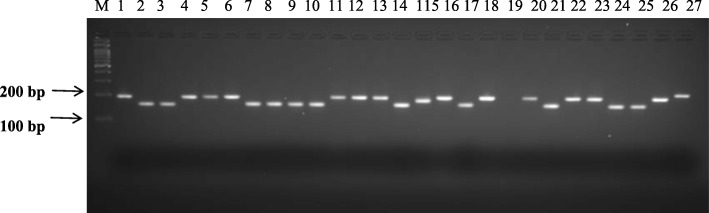
PCR amplification of genic-SSR marker TWSSR62 in 27 blackgram genotypes

In this study, 31 SSR primers were screened with 27 blackgram genotypes which yield 89 alleles with an average of 2.9 alleles/locus and the number of alleles varied from 1 to 5 with PIC ranging from 0.14 to 0.85, with a mean value of 0.54 (Table 1). Representative amplification of genic-SSR primer (TWSSR62) is shown in Fig. 4. Dendrogram generated based on blackgram genic-SSR markers distributed all 27 genotypes into one major and one minor cluster (Additional file 6: Figure S4). Among the 27 genotypes, DPU88–31 and IPU02–43 showed the highest similarity index, while the genotypes TU94–2 and LBG693 showed the lowest. The minor cluster comprised of LBG752, LBG709, LBG703 and LBG693. While the remaining 23 genotypes grouped into major cluster in which TW is represented as OTU.

**Table 1:** Number of alleles per locus and polymorphic information content (PIC) value of genic-SSR markers used for studying allelic variation among blackgram genotypes

Genic-SSR primer code	No. of alleles /locus	Polymorphic information content (PIC)	Putative function
TWSSR-1	2	0.28	Protein ABCI7
TWSSR-4	2	0.21	transcription factor bHLH143-like
TWSSR-8	3	0.76	CBL-interacting serine/threonine-protein kinase 8
TWSSR-9	3	0.62	*Vigna angularis* var. *angularis* DNA, chromosome 11
TWSSR-10	5	0.67	transcription factor TCP9
TWSSR-11	2	0.53	U-box domain-containing protein 4
TWSSR-12	4	0.49	*Vigna radiata var. radiata* uncharacterized LOC106765753
TWSSR-13	2	0.14	sugar carrier protein C
TWSSR-14	3	0.34	*Vigna angularis* var. *angularis* DNA, chromosome 1
TWSSR-15	3	0.60	vacuolar sorting receptor
TWSSR-16	4	0.50	*Vigna radiata* var. *radiata* uncharacterized LOC106778819
TWSSR-19	3	0.67	alpha,alpha-trehalose-phosphate synthase [UDP-forming] 1
TWSSR-20	4	0.72	transcription factor 25
TWSSR-24	1	0.14	glycerol-3-phosphate transporter 4
TWSSR-31	2	0.58	*Lupinus angustifolius* cultivar Tanjil chromosome LG-11
TWSSR-34	2	0.59	60S ribosomal protein L29–1
TWSSR-47	3	0.48	hypothetical protein (PHAVU_001G023500g)
TWSSR-48	2	0.50	Human DNA sequence from clone RP11-164 K15 on chromosome 22
TWSSR-57	2	0.48	vesicle-associated membrane protein 722-like
TWSSR-59	3	0.57	*Vigna angularis* var. *angularis* DNA, chromosome 4
TWSSR-61	3	0.85	glycine-rich domain-containing protein 1
TWSSR-62	4	0.69	transcription initiation factor IIF subunit beta
TWSSR-66	3	0.52	*Vigna radiata* var. *radiata* uncharacterized LOC106765616
TWSSR-68	3	0.79	UPF0496 protein At2g18630-like
TWSSR-72	2	0.46	*Vigna angularis* uncharacterized LOC108326918
TWSSR-74	4	0.77	ATP-dependent RNA helicase eIF4A
TWSSR-76	1	0.65	activator of 90 kDa heat shock protein ATPase homolog
TWSSR-81	1	0.36	trafficking protein particle complex subunit 6B
TWSSR-82	3	0.69	*Vigna angularis* var. *angularis* DNA, chromosome 8
TWSSR-86	3	0.54	serine/threonine-protein kinase-like protein At3g51990
TWSSR-87	3	0.46	histidine kinase 4
Average	3	0.54

### Identification and validation of RNA Seq data by qRT-PCR

A total of 2306 CDS were differentially expressed in both the samples, 1190 CDS were found to be down-regulated and 1116 CDS were found to be up-regulated with a mean fold change value of − 375 (Additional file 7: Table S3). Most of the differentially expressed genes belongs to signal transduction pathways. Expression pattern of 7 DEGs (5 up and 2 down-regulated CDS) was analysed through qRT-PCR. All 7 DEGs analysed, showed expression profile consistent/ similar to RNA Seq data but differed in expression magnitude (Additional file 8: Table S4).

### Identification and characterization of SNPs

**Fig. 5:**
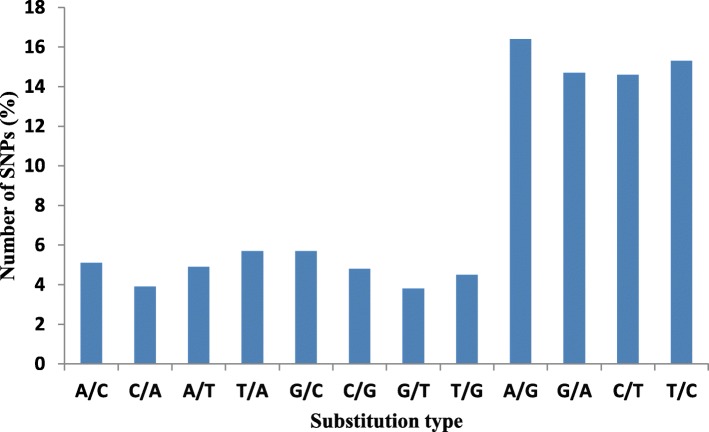
Frequency and substitution types of the identified SNPs of wild blackgram TCS

A total number of 1844 SNPs were identified in TW sample in which 17 SNPs were heterozygous and 1828 SNPs were homozygous. SNPs were filtered at a minimum read depth which varied from a minimum of 20 to a maximum of 200. Out of 1844 SNPs, 1291 (70%) occurs within CDS, 518 (28%) lie in untranslated regions (269,14.6% in 3′-UTR and 249,13.5% in 5′-UTR) and for 36 SNPs bearing TCS, no ORF was predicted by ORF finder (Fig. 3). All the SNPs were classified into 8 transversion and 4 transition substitution classes. The numbers of transition (Ts) and transversion (Tv) type of SNPs were 1129 (61%) and 716 (39%) respectively, with a Ts/Tv ratio of 1.57 for TW. Most of the SNPs (61%) falls under transition substitution class, in which most abundant are A/G (16.4%) type SNP followed by T/C (15.3%),G/A (14.7%) and C/T(14.6%) (Fig. 5). In case of transversion substitution class, the frequency of occurrence of the SNPs were as follows: T/A (5.7%) and G/C (5.7%), followed by A/C (5.1%), A/T (4.9%), C/G (4.8%), C/A (3.9%) and G/T (3.8%).

### Development of genic-SNP markers

Out of 1844 SNPs identified, PCR primer pairs were successfully designed for 1749 SNP loci. Details of primer sequence, expected product size and T_m_ is provided in tabular form (Additional file 9: Table S5). Primers could not be designed for the remaining 95 SNP loci because their flanking sequences were either too short or the nature of sequence did not fulfil the criteria for primer design. One hundred primer pairs flanking single SNP were selected for screening in TW and TU94–2 genomic DNA. Eighty five primer pairs successfully amplified in end-point PCR for both samples and 79 primer-pairs showed monomorphic pattern. These 79 primers were proceeded for SNP genotyping by HRM analysis.

### SNP genotyping by HRM assay

**Fig. 6:**
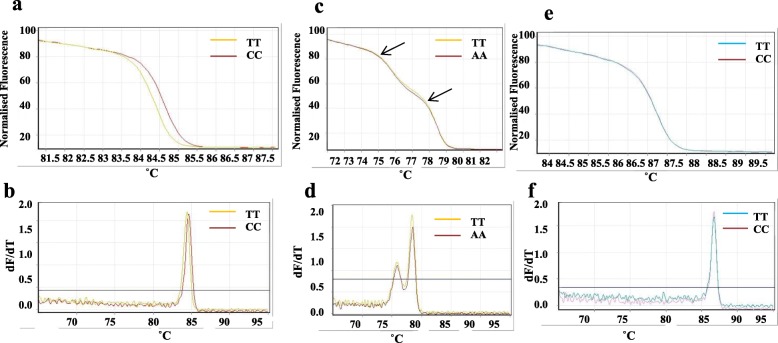
High resolution melting (HRM) curve profiles of three HRM amplicons. **a** normalised plot of two homozygous genotypes showing one melting domain, **b** derivative plot of a, **c** normalised plot of two genotypes heterozygous at one SNP site (T/A) showing two melting domains (arrows), **d** derivative plot of c, **e** normalised plot of two genotypes with no apparent SNP site (T/C) showing single curve, **f** derivative plot of e

A total of 71 out of the 79 SNPs were amplified through PCR-HRM cycling. Raw HRM profiles were selected by data quality control parameters and analysed through HRM analysis software. Fifty four primer pairs with C_T_ value ≤ to 30 and amplification efficiency > 1.4 fulfilled the quality control criteria were selected for further analysis. Melt curves, normalized graph (obtained from raw graph after normalization) and derivative plot was analysed with the help of default software. While the remaining 17 primer pairs which did not fit into criteria were also analysed. A total of 57 SNPs (78.8%) were validated as homozygous by HRM assay which were also predicted to be homozygous through transcriptome sequencing. These validated SNPs showed single melting transition in normalized HRM melt curves and single narrow peak in derivative plot. Twelve SNPs showed aberrant curves with multiple melting phases or peaks characteristic of heterozygosity and melting temperature difference between both the genotypes was not detected for 3 SNPs. Representative SNPs showing homozygous and heterozygous pattern for normalized HRM curve and derivative plot are shown in Fig. 6. No primer-dimers and non-targeted amplicons were seen for HRM PCR products when resolved on agarose gel (Additional file 10: Figure S5).

### Melting temperature difference, genotypes and SNP class

For SNPs in class I, II and III, homozygous wild-type and homozygous mutant samples were easily distinguished from each other by a shift in T_m_. However, the T_m_ difference between homozygous genotypes for SNPs in class 4 was smaller than in class I, II and III. Minimum T_m_ difference seen for class IV SNP is 0.02. In this study, 31 SNPs come under class I SNP with a T_m_ difference range of 0.15 to 0.51, 15 SNPs appear to fall in class II SNPs with a T_m_ difference range of 0.03 to 0.41, 2 and 8 SNPs fall into class III and IV respectively, with their corresponding T_m_ difference range of 0.18 to 0.35 and 0.02 to 0.12.Three SNPs (TWSNP 76, 85 and 121) showed no T_m_ difference. In the present study, one complex amplicon bearing 2 SNPs i.e. TWSNP 50 (T/A and A/G) was genotyped correctly with HRM as homozygous for both positions. In this study, class I and IV SNPs can be distinguished by HRM analysis based on T_m_ difference while other classes are difficult to differentiate from each other due to overlapping T_m_ difference.

### Functional significance of SNPs validated by HRM analysis

Among SNP loci validated by HRM analysis, 13 SNP loci found to be non-synonymous missense substitutions and thus are expected to have affected the encoded proteins. These 13 SNPs found in a cytochrome c-type biogenesis protein CcmE, U-box domain-containing protein 13, cytochrome P450, pre-mRNA-splicing factor ATP-dependent RNA helicase DEAH2, protein phosphatase, mitochondrial fission 1 protein A-like, dnaJ protein homolog 1, cell division cycle protein 48 homolog, lariat debranching enzyme, 2 uncharacterized proteins and 2 hypothetical proteins - resulted in missense substitutions and amino acid replacements (D to N, E to D, N to K, K to R, V to F, A to T, M to I, T to I, R to Q, T to A, N to D, I to V and K to N respectively). ITASSER results revealed alteration in secondary structure of encoded proteins due to missense mutations. The variant form in contrast to native form of five proteins - U-box domain-containing protein 13, pre-mRNA-splicing factor ATP-dependent RNA helicase DEAH2, uncharacterized protein LOC108329963 [*Vigna angularis*], lariat debranching enzyme and cell division cycle protein 48 showed changes in protein secondary structure (Additional file 11: Table S6). For U-box domain-containing protein 13, pre-mRNA-splicing factor ATP-dependent RNA helicase DEAH2 and cell division cycle protein 48 changes in secondary structures do not resulted in changes in ligand / substrate binding preference or ligand binding sites. But for uncharacterized protein LOC108329963 [*Vigna angularis*] and lariat debranching enzyme changes in number of helices and strands resulted in alteration in preferred ligand. However, for six genes -cytochrome c-type biogenesis protein CcmE, uncharacterized protein LOC108329961 [*Vigna angularis*], cytochrome P450, hypothetical protein LR48_Vigan10g050400 [*Vigna angularis*], hypothetical protein VIGAN_01393500 [*Vigna angularis* var. *angularis*], dnaJ protein homolog 1, number of helices and strands predicted by protein modelling for native and variant form of the protein were same but the preferred ligand changed. For example, native form of cytochrome P450 binds preferably to ligand zinc but variant form binds with calcium. While for remaining two genes- protein phosphatase 2C 55 and mitochondrial fission 1 protein A-like, missense mutations do not altered neither secondary structure nor preference for the ligand.

### Validation of target SSR motif and SNP amplification through re-sequencing

PCR products amplified using five SSR primers TWSSR 13, 57, 62, 86 and 96 were re-sequenced which revealed the presence of repeat sequence (GAA)_10_ (AG)_8_ (TC)_19_, (GAT)_11_ (TCA)_6_ respectively. This is similar to the original transcriptome sequence which indicated the successful target amplification. HRM PCR products were sequenced to confirm the HRM assay results. Sixteen amplicons were re-sequenced and 9 were found to be homozygous similar to transcriptome sequencing. Remaining SNPs were could not be confirmed due to sequencing error.

## Discussion

Transcriptome sequence data of immature seeds of wild blackgram would help in understanding the gene expression pattern underlying the seed development and provides a scope to develop gene-based markers. Transcriptome dataset will be useful resource to incorporate desirable improvements for seed quality and yield [35] and can function as a reference for studying effects of biotic, abiotic stresses, specific mutations on phenotypes [36]. Moreover, wild accession of blackgram, *Vigna mungo* var*. silvestris* is highly relevant to the breeding programme as a source of bruchid resistance genes. Transcriptome sequencing of cultivated [37] and wild blackgram species (this study) helps in revealing the genetic basis of differences between both species and other aspects of genome evolution. Transcriptome sequencing of cultivated and wild species has been reported for some crops including soybean [38–41] chick pea [33, 42–44], pigeonpea [45], mungbean [46], adzuki bean [47] peanut [34], wheat [48] and *E. sibiricus* [49].

### Wild blackgram transcriptome characterization

Transcriptome analysis is a better alternative for understanding the functional complexity of genomes for plant species with large genome size which presents a great challenge for whole genome sequencing [50].The objective of this project was to assemble a reference transcriptome from immature seeds, followed by its characterisation and development of marker resources. In this study, ~ 40,178 transcript contigs (TCS) were obtained which is less than 48,291 for the cultivated blackgram. This could be attributed to the smaller seed size of the wild species, difference in gene expression pattern, or due to introgression or loss of genes in the cultivated blackgram during its domestication and technical errors during sample processing. The length of contigs ranged from 280 to 6445 bp with an average length of 446 bp is comparable to that reported for cultivated blackgram [37]. Blackgram wild accession CDS showed 27% similarity to common bean followed by 16% similarity with *Citrus clementina* and 7% similarity to *Glycine max* although cultivated TU94–2 genotype CDS showed 50% similarity to common bean followed by 11% with *Glycine max* [37]. Above observations indicated homology among the wild, cultivated blackgram and common bean as they belong to sub-tribe Phaseolinae. Similar gene conservation has been reported within legumes such as field pea, faba bean, lentil with *Glycine max* [50, 51] and chickpea with *Medicago*, soybean and common bean [52]. Around 25.20% CDS of wild species did not show any hit in the database which could be composed of alternative splicing variants, novel genes or differentially expressed genes [53], crop-specific genes [33, 42, 54] and unique contigs with less conserved regions such as 3′-UTRs; C-termini or 3′ sequences [55]. Gene Ontology terms were assigned to 72% of the total CDS to known gene functional distribution which is more as compared to 65% for cultivated because TW being a wild genotype is expected to be more genetically diverse. Among the 140 KEGG pathways identified for TW, 524 CDS were fall under purine metabolism which played role in ammonia assimilation and detoxification in specialized tissues [37], primary nitrogen metabolism in tropical legumes such as soybean [56], nucleotide biosynthesis and degradation in cotyledons and embryonic axes of blackgram [44]. Annotation of the CDS using these methods help in predicting potential genes and their diverse functions as reported for lentil transcriptome [50].

### Frequency and distribution of genic-SSRs

In this study, 1621 genic -SSRs were identified in 3.3% of the transcripts with a frequency of one SSR per 11.1 kb which is in accordance with our report for blackgram cultivated variety [37] but higher as compared to 3.3 kb in mungbean [57], 8.4 kb in pigeonpea [45] and 7.4 kb in soybean [58].The less number of SSRs for wild species, may be accounted for less number of transcripts (40,178) as compared to cultivated species (48,291). The frequency of SSR in different plant species varies [59, 60] depending on genomic composition and direct comparison among different crops is difficult due to SSR search criteria, size of the dataset analysed, database-mining tools and sequence redundancy [61].

In the present study, tri-nucleotides were found to be the most abundant for blackgram wild species (39.9%). Similar trend was reported in lentil [50], mungbean [57], chickpea [62], peanut [19] and common bean [5]. Such dominance of triplets over other repeats in coding regions may be due to the suppression of nontrimeric SSRs in coding regions as they may lead to frame-shift mutations [63]. Among tri-nucleotides, AAG/CTT (29.9%) was found to be the most frequently occurring motif, which was consistent with cultivated blackgram [37], cultivated peanut [19], chickpea [33, 42], mungbean [46], adzuki bean [11] and pigeonpea [45]. Dinucleotide (30.2%) repeats were the second most abundant in TCS similar to cultivated blackgram. AG repeats (AG/CT class) were by far the most common dinucleotide repeat, constituting nearly 66% of dinucleotide repeats which is similar to that found in plants including legumes like chickpea (71.1%), blackgram (59.7%) [7, 33]. In this study, sequence analysis revealed that di-nucleotide repeats were almost equally associated with UTRs (235, 48%) and CDS (242, 49.4%) regions, whereas tri-nucleotides repeats (444,68.7%) were preferentially present within CDS. The 5’UTRs contained more triplets than the 3’UTRs, similar observation was reported for blackgram [37]. SSR distribution in the coding regions, UTRs and introns is non-random, vary among species and/or taxonomic groups and is strongly biased as it is a function of relative frequencies of replication slippage, point mutation, selection pressure and environmental stresses that increase mutation frequency in SSRs [60, 64–66]. Genic-SSR markers based on transcriptome dataset has been developed for many legume species including blackgram [37], mungbean [57], cowpea [67], chickpea [43, 62], pigeonpea [45], common bean [5], chickpea [28]. In this study, 1171 primer pairs were designed for SSRs from the transcriptome dataset from wild species. One hundred SSR primers were tested for amplification. Of which, 58 primer pairs showed amplification. Low amplification rate observed in this study could be due to sequencing errors or occurrence of SNPs causes disrupting of priming sites and large PCR amplicons due to intervening introns.

### Genetic diversity analysis in blackgram germplasm

Allelic variation at 31genic-SSR loci produced 89 alleles in 27 blackgram accessions and PIC value varied from 0.14 to 0.85 with an average of 0.54 which is comparable to other legumes including cowpea [68, 69] and pigeonpea [45] and higher than cultivated blackgram. PIC also depends on sample size analysed, accession source and marker scoring skills. High PIC values observed in this study suggests the efficacy and utility of the developed markers. In the present study, null alleles were observed for nineteen SSR primers among different blackgram genotypes studied which may be due to primer site variation or unrecognized intron splice sites disrupting priming sites or presence of large introns between the primers, resulting in a too large product or in extreme cases, failed amplification [37].

### Frequency and distribution of genic-SNPs

EST based SNP has been used for high-resolution genotyping, construction of genetic maps, and genome-wide association studies in agricultural crops [28]. Using stringent criteria, we identified 1845 SNPs distributed in 40,178 TCS of TW. The frequency of transitions and transversions were comparable to that observed in chickpea [28], soybean [70], mungbean [71]. Transition (T_s_) to transversion (T_v_) ratio was calculated to understand the process of molecular evolution [72] and assessing the quality of SNP calls [73] The transition to transversion ratio was 1.6 in TW, which is a high ratio compared to that in maize [74]. A higher Ts/Tv ratio generally indicates higher accuracy [75]. Transition bias over transversion has been considered universal in the genome [63, 76] and considered to be partially due to cytosine methylation [77]. Therefore, the high transition bias may reflect high methylation levels in the blackgram genome.

### HRM assay

HRM is a feasible means for SNP genotyping which is useful in plant cultivar identification, genetic mapping, QTL analysis, diagnosis of pathogenic species, and gene discovery [22] and has reported applications in plants such as alfalfa [78], almond [22], potato [79] and apple [80]. In this study, we attempted to identify and validate SNPs through HRM assay. A total of 71 SNPs out of 79 pre-screened SNPs through endpoint PCR were successfully amplified in HRM-PCR programme. In this study, we genotyped SNPs with a validation rate of 78.87% which is close to other HRM assay reports such as 91% for alfalfa [81] although validation rate depends on amplicon size, number of SNPs genotyped and varies from study to study. In addition, failed PCR amplification due to disrupted primer sites or large amplicons due to intervening introns (high Ct value) also affects considerably to validation rate. Notably with standardization of kit components, primer concentration, template DNA concentrations, *in-silico* detection of amplicon and primer-pairs for secondary structures, proportion of SNPs validated can be increased and assay can be rapidly applied for plant SNP identification and validation accurately with its low cost, simple and high-throughput features. Twelve SNPs genotyped through HRM analysis appeared as heterozygous did not match the inferred transcriptome sequence were assigned as putative homozygotes. Similar observations were reported in alfalfa [81]. Occurrence of multiple melt domains in HRM curve is not only because of heteroduplex formation from heterozygous genotypes, but could be due to sequencing and experimental errors, secondary structures, characteristics of DNA, concentrations of the ions and the volume of the solution in the assay system [22] and sensitivity or specificity of HRM machine.

A/T substitution have been reported as the most difficult SNP class to be resolved by melting analysis [65, 82]. In this study, the genotypes with A/T variation (8 TWSNPs) were distinctly differentiated from class I SNP. SNP genotyping by high-resolution melting is simple and cost effective as only PCR primers and a generic dye are needed with no need for probes [65]. Seventeen SNPs which did not obey quality control parameters were also analysed through normalized HRM curves. Of which, 10 SNPs were genotyped as homozygous by HRM assay which is in accordance with the transcriptome sequencing results and 7 SNPs showed multiple melt domains. This suggests that HRM melt profiles with single melting transtition can be genotyped for zygosity regardless of data quality control parameters. To our knowledge, this is the first report of developing cost effective HRM assay for genotyping and validating SNPs identified from transcriptome sequencing in blackgram which will enhance molecular breeding.

### Functional significance of SNPs validated by HRM analysis in different genes

Detection of thirteen non-synonymous SNP loci showing missense mutations in important physiological genes (cytochrome c-type biogenesis protein, U-box domain-containing protein 13,cytochrome P450, pre-mRNA-splicing factor ATP-dependent RNA helicase DEAH2, protein phosphatase, mitochondrial fission 1 protein A-like, dnaJ protein homolog 1 and cell division cycle protein) that differentiated the wild accession from TU94–2 variety could help in differentiating the wild accession from other cultivars for their differential reaction to several stimuli. Moreover, such non-synonymous SNPs in genes that affect the structure and function of encoded proteins to generate favourable alleles providing adaptive and evolutionary advantages [83]. A non-synonymous SNP in U-box domain-containing protein 13 could explain the tolerance of plant towards various abiotic stresses such as cold, heat, salt. U-box containing genes are extensively studied for their role in abiotic stresses in *Arabidopsis* [84]. Role of pre-mRNA-splicing factor ATP-dependent RNA helicase DEAH2 which is involved in RNA processing was studied in *Arabidopsis* and found to be associated with early flowering [85]. Cytochrome P450s are abiotic stress-responsive genes and reported to be involved in the biosynthetic pathways of plant allelochemicals such as insect toxins and repellents [86]. Therefore, a non-synonymous SNP altering secondary structure and thus catalytic activity could be responsible for difference in susceptibility to various insects found in crop germplasm. Similarly, the role of HSP40/Dnaj protein in viral pathogenesis has been well studied in various virus-plant interactions such as for *Potato virus Y* [87] and *Tomato spotted wilt virus* [88]. Thus detection of a non-synonymous SNP could aid in developing molecular marker linked to these virus resistance genes. Protein phosphatase 2c is known to be involved in abscisic acid (ABA) signal transduction and thus presence of a non-synonymous SNP can alter protein secondary structure and probably its function thus affecting plant growth, seed dormancy and stomatal closer as studied in *Arabidopsis* [89]. Similarly, cell division cycle CDC48 regulates the turnover of immune receptors and mediates the degradation of viral proteins in plant cells [90].

## Conclusions

In the present study, 1171 genic-SSR and 1749 SNP markers were generated in large-scale by Illumina paired end sequencing of wild blackgram immature seeds. Genetic relationship among 27 blackgram genotypes were studied using selected genic-SSR primers. The distribution and characteristics of SNP markers in blackgram were evaluated for the first time using transcriptome dataset from wild blackgram accession. Moreover, this is the first attempt to develop HRM assay to validate SNPs experimentally in blackgram. The availability of such a large number of sequence-based markers allows genetic diversity analysis, linkage mapping, comparative genomics, and association studies. The data generated in the current study such as newly identified SSR and SNP markers and DEGs provide a valuable resource for genetic enhancement of blackgram through marker assisted breeding.

## Methods

### Sample preparation, transcriptome sequencing and De novo assembly

The wild accession of blackgram, TW (*Vigna mungo* var*. silvestris*) was used for generating transcriptome dataset in this study. Total RNA was extracted from 12 immature seeds collected 4 weeks after flowering of different TW plants using RaFlextotal RNA isolation kit as per protocol. The quality and quantity of the isolated RNA samples were assessed by QubitFluorometer. The paired-end cDNA sequencing libraries preparation was done with the help of IlluminaTruSeq RNA Sample Preparation V2 kit. For Library preparation mRNA was fragmented, reverse transcribed, second-strand synthesized, ligated with paired-end adapters and index PCR amplification of adaptor-ligated library was done. Quantity and quality of library was checked on Agilent Caliper LabChip GX Bioanalyser using DNA High Sensitivity Assay Kit. Sequencing was performed in a single lane in Illumina MiSeq using paired end sequencing chemistry (Xcelris Genomics Ltd. Ahmedabad). The raw data was filtered for adaptor contamination and low quality value reads i.e. an average QV less than 20 (QV < 20) and these reads were removed with the help of Trimmomatic v0.30 [91]. Assembly of high quality reads were performed with CLC Genomics Workbench on default parameters (minimum contig length: 200, automatic word size: yes, perform scaffolding: yes, mismatch cost: 2, insertion cost: 3, deletion cost: 3, length fraction: 0.5, similarity fraction: 0.8). Bioinformatic analysis were performed with the assembled transcript contigs.

### Coding sequences (CDS) prediction and functional annotation

Identification of CDS was carried out using ORF-predictor and the longest frame out of the six frames was selected. A total of 38,753 CDS were predicted which were further annotated using BLASTx. The functional annotation was performed for 38,753 CDS by similarity search program, BLASTx which aligned the CDS to non-redundant protein sequence (nr) database of NCBI (http://www.ncbi.nlm.nih.gov) against green plant database. The transcripts with significant similarity (e-value ≤1e-5) were assigned the putative function as that of corresponding protein from green plant database and mapped on Gene Ontology (GO) database to classify based on their functions. GO mapping was carried out using Blast2GO [92]. CDS were mapped to their respective pathways with the help of KEGG (Kyoto Encyclopedia of Genes and Genomes) database [93] (http://www.genome.jp/kegg).

### Identification of SSRs and primer designing

SSRs were searched using WebSat (http://purl.oclc.org/NET/websat/) online software [94]. SSR motifs with two to six nucleotides in size and a minimum of 6, 5, 4, 4, 3 contiguous repeat units for di-, tri-, tetra-, penta- and hexa-nucleotide respectively were considered for this study. Mononucleotide repeats were excluded from the search criteria. After SSRs searching, primers were designed using WebSat (http://purl.oclc.org/NET/websat/) online software [94] with following parameters: optimum primer length of 22 mer (range: 18–27 mer), optimum annealing temperature at 60 °C (range: 57–68 °C), GC content ranged from 40 to 80% while other parameter values set as default. Hundred SSR primer pairs (synthesized by Eurofins Genomics) were randomly selected for screening in four blackgram varieties and predicted polymorphism was further validated in 27 blackgram genotypes. SSRs frequency of occurrence and relative abundance were analysed and expressed as SSR per kb of sequence. Classification of SSRs on the basis of their position in the coding sequence (CDS) or untranslated region (5′ or 3′ UTR) of the gene was done. The transcripts were analyzed for ORFs with the help of ORF Finder software (http://www.ncbi.nlm.nih.gov/gorf/gorf.html) and the longest one starting with ATG codon was selected as protein encoding transcript.

### Plant material, DNA extraction and SSR amplification

Wild accession of blackgram TW (*Vigna mungo* var. *silvestris*) is a native of Trombay hills and is maintained at Nuclear Agriculture and Biotechnology Division, Bhabha Atomic Research Centre (latitude 18:54 N, longitude 72:49E), Mumbai, India and do not required any permissions or licenses whereas other cultivated accessions were taken from the Institute germplasm collections. Twenty eight blackgram genotypes used in this study are described in Additional file 12: Table S7. Total genomic DNA was extracted from two-day old seedlings by Dellaporta method [95]. The quality and quantity of extracted DNA samples were assessed by Nanodrop ND 1000 spectrophotometer (Thermo Scientific, USA). one hundred SSR primers were pre-screened in 6 blackgram genotypes. Selected 31 SSR primers were used for genetic diversity analysis among 27 blackgram genotypes. For SSRs, PCR reactions were carried out in a 25 μl reaction as per the reported cycling conditions [37]. PCR products were resolved on 3% agarose gels in TBE buffer at 80 V, stained with ethidium bromide and the image captured in a gel documentation system.

### Diversity analysis

Polymorphic markers were scored for each SSR allele as presence (1) or absence (0) of amplified products. Allelic variation among blackgram genotypes were analysed by calculating the polymorphic information content (PIC) of each SSR marker by applying the formula of Anderson et al. [96]: PIC = 1 − S(P_*ij*_)2, where P_*ij*_ is the frequency of the *j*^th^ allele for the *i*^th^ locus. Dendrogram was generated based on Jaccard’s similarity coefficients which were subjected to the unweighted pair group method with arithmetic average (UPGMA) using software NTSYS-pc version 2.0 [97].

### Identification of differentially expressed genes (DEGs) and primer designing

For identifying DEGs, TU94–2 was assigned as control and Trombay wild as treated. The high quality reads for each samples were mapped on to their respective set of CDS using CLC and FPKM was calculated using the formula: FPKM = 109 x C/ (N x L), where, C is the number of reads mapped onto the transcript contigs, N is the total number of mappable reads in the experiment, L is the number of base pairs in the transcript contigs. After FPKM calculation, common hit accessions were found based on BLAST against Green Plant database for DEGs. CDS were further classified as up and down regulated based on their log fold change (FC) value calculated by the formula: FC = Log2 (Treated/Control). FC value greater than zero were considered up-regulated whereas less than zero were down-regulated.

### Validation of RNA Seq data by qRT-PCR

Primers were designed for randomly selected 7 DEGs from Trombay wild CDS with the help of Gene Runner v. 5.0.99 Beta software (Hastings Software, Inc.).Total RNA extraction from both Trombay wild and TU94–2 variety were performed with Spectrum™ Plant Total RNA Kit (Sigma-Aldrich, USA) and treated with DNAse-I (Sigma-Aldrich, USA) to eliminate traces of genomic DNA. The cDNA was synthesized using a PrimeScript™ RT-PCR Kit (Clontech, USA) and real time PCR protocol was followed according to manufacturer’s instructions given in SYBR1 Premix ExTaq™ (Tli RNAse H Plus) (Clontech,USA). The thermal cycling conditions were carried out in Rotor-Gene-Q Real-Time PCR System (Qiagen, USA) using the following program- 95 °C for 5 min followed by 40 cycles of 94 °C for 30 s, 62 °C for 20 s and 72 °C for 20 s followed by melting PCR products from 65 °C to 95 °C. The relative gene expression levels were calculated by REST software [98].

### Identification of SNPs

SNPs were discovered by alignment of high quality reads (HQ) of TW as alternate with the assembled transcript of TU94–2 sample as reference. HQ-reads were mapped on the assembled transcripts with the help of BWA version 0.7.5a with default parameters and alignment results were generated in SAM (Sequence Alignment/Map) format which were further converted into BAM (binary version of a SAM file) format using samtools and sort programs. These sorted BAM files were used to call SNPs using mpileup program of samtools using default parameters and varFilter program of bcftools with default parameters except minimum read depth, maximum read depth and mapping quality were assigned to 20, 200 and 10 respectively. The SNPs were obtained in vcf (Variant Call Format) file.

### Primer designing, characterization of SNPs and functional significance

Primers were designed for all SNPs of TW based on TU94–2 transcripts available from NCBI (submission ID. SRR 1616991, SRX710526 and study accession SRP 047502**)** with the help of Primer3 software [99, 100] (http://sourceforge.net/projects/primer3/)**.** For designing PCR primers, optimum primer length was 20 mer (range: 18–27 mer), optimum annealing temperature was 60 °C (range: 57–63 °C), GC content ranged from 20 to 80%, PCR product size ranged from 100 to 250 bp and other parameter values as default. All 1844 SNPs bearing TU94–2 TCS were analysed for the presence of ORF regions and classified into three classes, i.e., whether lying in 5’UTR, CDS or 3’UTR region. SNPs were also characterised into transition and transversion classes. Transition (T_s_) to transversion (T_v_) ratio was calculated as reported by Sablok et al. [72].

To know the functional annotation, SNP contigs were searched for homology with other proteins in NCBI through BLASTn algorithm. Amino acid sequences encoded by the coding nucleotide regions of non-synonymous SNPs were analyzed further using the ITASSER automated web server [101] for prediction of ab intio three dimensional secondary protein structure and active catalytic domain binding sites with ligands. The high quality protein model of correct topology was selected based on high confidence scores.

### Realtime HRM-PCR amplification and data analysis

HRM assay was performed in Rotor-Gene Q realtime PCR Thermocycler (Qiagen) using pre-screened amplified primers as per the protocol of Type-it HRM PCR Kit (Qiagen). Specific amplification for each primer pair was checked on agarose gel and by melting analysis in Rotor-Gene Q. Raw HRM data was analysed with the help of inbuilt software in Rotor-Gene Q. For data quality control, PCR amplification was analysed through the assessment of the C_T_ value and amplification efficiency [22]. The amplification data which deviate from any of the two following criteria were not used for HRM analysis. HRM runs with CT value ≤30 with amplification efficiency > 1.4 were considered for analysis. Raw HRM curves were analysed using the HRM analysis module as per Corbett Research manual. Realtime PCR HRM amplicons were resolved on 3% agarose gels to detect the presence of primer-dimers, non-targeted amplicons. Melting temperature (T_m_) difference was calculated manually with the help of negative derivative plots. Homozygotes melt in a single transition whereas, heterozygotes showed multiple melt phases and the order of melting was correctly predicted by nearest-neighbor calculations as A/A _ T/T _ C/C _G/G [65]. Class 1 SNPs are C/T and G/A transitions that produce C::G and A::T homoduplexes and C::A and T::G heteroduplexes, class 2 SNPs (C/A and G/T) are transversions that produce C::T and A::G heteroduplexes, Class 3 SNPs (C/G) produce C::G homoduplexes with C::C and G::G heteroduplexes and Class 4 SNPs (A/T) produce A::T homoduplexes with A::A and T::T heteroduplexes [65]. SNPs were differentiated into 2 groups, homozygous and heterozygous based on normalised HRM curve. SNP amplicon sequences were checked for the presence of SSRs with the help of WebSat online software.

### Validation of SSRs and HRM results through re-sequencing

Successful amplification of target SSR sequence was validated through re-sequencing of PCR products. For this, PCR products amplified with 5 SSR primers in two genotypes were re-sequenced. A total of 16 SNPs were selected for confirming HRM results by sequencing PCR products of both TU94–2 and TW genotypes. Based on the HRM results, five SNPs genotyped as homozygous and another five representative SNPs from each different type of HRM melt curves and derivative plots were chosen for sequencing. PCR products of both genotypes were re-sequenced on ABI platform (APS Labs). Forward and reverse (reverse complementary) reads of both genotypes were aligned with the TU94–2 contigs through Clustal Omega online multiple sequence alignment tool.

## Additional files

Additional file 1: **Figure S1.** Length-wise distribution of transcript contigs of blackgram transcriptome. (DOC 55 kb)

Additional file 2: **Figure S2.** Blast hit distribution of wild blackgram CDS similar to known proteins of non-redundant (nr) database resulted from BLASTx algorithm with an E value threshold of 10^− 5^ available at NCBI. (DOC 41 kb)

Additional file 3: **Table S1.** Gene Ontology (GO) classification of the predicted CDS. (XLS 1970 kb)

Additional file 4: **Figure S3.** Distribution of wild blackgram CDS based on ontologies: molecular function, biological process and cellular component. (DOCX 13 kb)

Additional file 5: **Table S2.** Genic-SSR primers designed from transcriptome sequence of wild blackgram. (XLSX 100 kb)

Additional file 6: **Figure S4.** Dendrogram showing the genetic relationship among 27 blackgram genotypes. (DOCX 38 kb)

Additional file 7: **Table S3.** Details of differentially expressed genes (DEGs) identified by RNA Seq between wild and cultivar accessions of blackgram. (XLS 750 kb)

Additional file 8: **Table S4.** Details of CDS sequences (Trombay wild genotype) taken for validating RNA Seq data by qRT-PCR. (DOCX 16 kb)

Additional file 9: **Table S5.**Genic-SNP primers designed from transcriptome sequence of wild blackgram. (XLSX 155 kb)

Additional file 10: **Figure S5.** PCR HRM products of TW and TU94–2 genotypes for TWSNP markers were resolved on 3% agarose gel. (DOCX 169 kb)

Additional file 11: **Table S6.** Non-synonymous SNPs validated by High Resolution Melting (HRM) analysis in TU94–2 and Trombay wild accessions of blackgram. (DOCX 17 kb)

Additional file 12: **Table S7.** Details of blackgram genotypes used in this study. (DOCX 14 kb)

## Data Availability

The datasets generated and / or analysed during the current study are available in the NCBI Short Read Archive (SRA) repository (http://www.ncbi.nlm.nih.gov/sra/) under the accession number SRP115376. The other supporting data were included as additional files.

## Abbreviations

CDS: Coding sequence
GO: Gene ontology
HRM: High Resolution Melting
KEGG: Kyoto Encyclopedia of Genes and Genomes
SNP: Single nucleotide polymorphism
SSR: Simple sequence repeats
UTR: Untranslated region
WT: Wild-type
