# Supplementary figures and images for: Transcriptome sequencing, de novo assembly, characterisation of wild accession of blackgram (Vigna mungo var. silvestris) as a rich resource for development of molecular markers and validation of SNPs by high resolution melting (HRM) analysis
Source: BMC Plant Biol. 2019 Aug 16;19:358. doi: 10.1186/s12870-019-1954-0 (PMC6697964; doi:10.1186/s12870-019-1954-0)

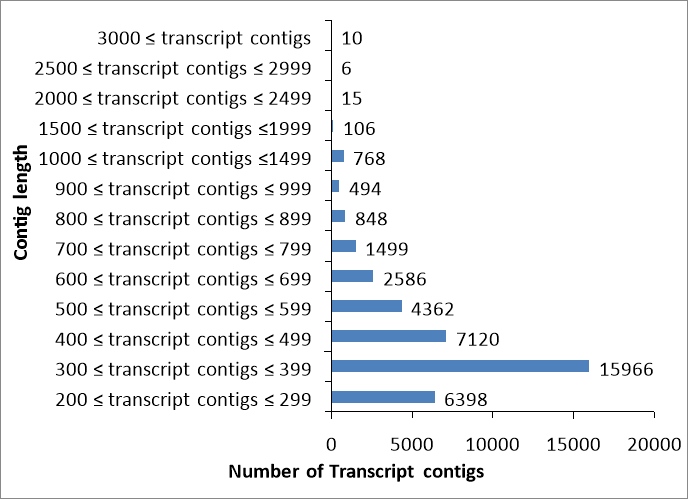

Supplement: Supplementary file 1 — Figure S1. Length-wise distribution of transcript contigs of blackgram transcriptome. (DOC 55 kb) [file 12870_2019_1954_MOESM1_ESM.doc]

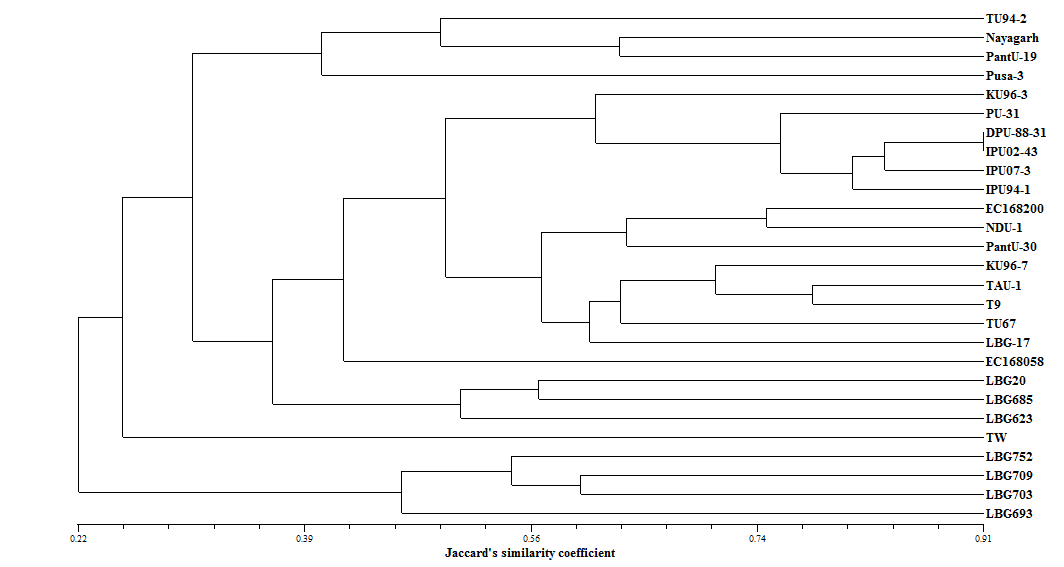


### 

Supplement: Supplementary file 6 — Figure S4. Dendrogram showing the genetic relationship among 27 blackgram genotypes. (DOCX 38 kb) [file 12870_2019_1954_MOESM6_ESM.docx]

**M 1 2 1 2 1 2 1 2 1 2 1 2 1 2 1 2 1 2**


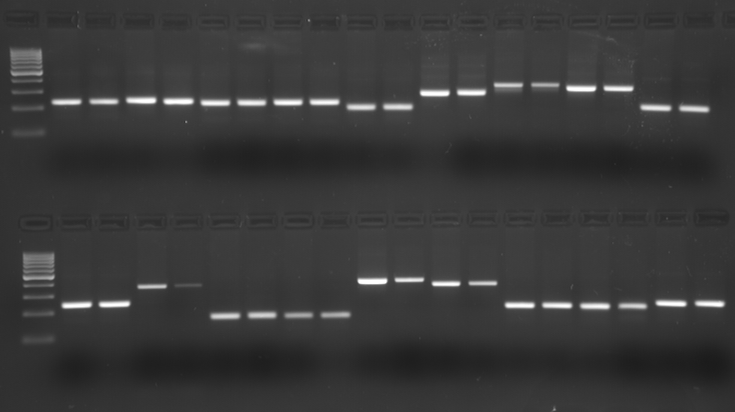


**300 bp**

**200 bp**

**100 bp**

**300 bp**

**200 bp**

**100 bp**

Supplement: Supplementary file 10 — Figure S5. PCR HRM products of TW and TU94–2 genotypes for TWSNP markers were resolved on 3% agarose gel. (DOCX 169 kb) [file 12870_2019_1954_MOESM10_ESM.docx]
