# Supplementary material for: Transcriptome sequencing, de novo assembly, characterisation of wild accession of blackgram (Vigna mungo var. silvestris) as a rich resource for development of molecular markers and validation of SNPs by high resolution melting (HRM) analysis
Source: BMC Plant Biol. 2019 Aug 16;19:358. doi: 10.1186/s12870-019-1954-0 (PMC6697964; doi:10.1186/s12870-019-1954-0)
Supplement: Supplementary file 8 — Table S4. Details of CDS sequences (Trombay wild genotype) taken for validating RNA Seq data by qRT-PCR. (DOCX 16 kb) [file 12870_2019_1954_MOESM8_ESM.docx]

| **CDS Id (Trombay wild)** | **Log fold change**  **obtained from RNA Seq** | **Log fold change**  **obtained from RT-PCR** | **Annotation by RNA Seq** | **Annotation through GO** | **Annotation through NCBI** |
| --- | --- | --- | --- | --- | --- |
| 5677 | 8.617372401 | 2.013 | gi\|561021812\|gb\|ESW20583.1\|hypothetical protein PHAVU_006G221200g [Phaseolus vulgaris] | acetylglucosaminyltransferase activity; | [PREDICTED: Vigna radiata var. radiata uncharacterized LOC106780510 (LOC106780510), transcript variant X18, misc_RNA](https://blast.ncbi.nlm.nih.gov/Blast.cgi#alnHdr_1255111182) |
| 16109 | 7.351490298 | 1.67 | gi\|561023383\|gb\|ESW22113.1\|hypothetical protein PHAVU_005G128300g [Phaseolus vulgaris] |  | [PREDICTED: Vigna radiata var. radiata protein RETICULATA-RELATED 1, chloroplastic (LOC106758785), mRNA](https://blast.ncbi.nlm.nih.gov/Blast.cgi#alnHdr_1255137576) |
| 124 | 5.134370622 | 0.033 | gi\|19911209\|dbj\|BAB86931.1\|glucosyltransferase-13 [Vigna angularis] | transferase activity, transferring hexosyl groups; | PREDICTED:Vigna radiata var. radiata hydroquinone glucosyltransferase-like (LOC106772886),mRNA |
| 21051 | 9.025439177 | 1.981 | gi\|561032294\|gb\|ESW30873.1\|hypothetical protein PHAVU_002G189500g [Phaseolus vulgaris] | organic cyclic compound binding; heterocyclic compound binding; | PREDICTED:Vigna radiata var. radiata glycine-rich RNA-binding protein 4, mitochondrial (LOC106767178), mRNA |
| 19831 | 6.934308158 | 2.431 | gi\|561012421\|gb\|ESW11282.1\|hypothetical protein PHAVU_008G016600g [Phaseolus vulgaris] |  | PREDICTED:Vigna radiata var. radiata golgin subfamily A member 6-like protein 6 (LOC106758498), mRNA |
| 3508 | -7.819903942 | -0.506 | gi\|561020039\|gb\|ESW18810.1\|hypothetical protein PHAVU_006G072200g [Phaseolus vulgaris] >gi\|561020040\|gb\|ESW18811.1\| hypothetical protein PHAVU_006G072200g [Phaseolus vulgaris] | ligase activity; small protein activating enzyme activity; ATP binding; |  |
| 25195 | -12.7884099 | -2.341 | gi\|115456709\|ref\|NP_001051955.1\|Os03g0857400 [Oryza sativa Japonica Group] >gi\|30102983\|gb\|AAP21396.1\| unknown protein [Oryza sativa Japonica Group] >gi\|108712199\|gb\|ABF99994.1\| expressed protein [Oryza sativa Japonica Group] >gi\|113550426\|dbj\|BAF13869.1\| Os03g0857400 [Oryza sativa Japonica Group] >gi\|215704395\|dbj\|BAG93829.1\| unnamed protein product [Oryza sativa Japonica Group] |  |  |
