# Supplementary material for: Transcriptome sequencing, de novo assembly, characterisation of wild accession of blackgram (Vigna mungo var. silvestris) as a rich resource for development of molecular markers and validation of SNPs by high resolution melting (HRM) analysis
Source: BMC Plant Biol. 2019 Aug 16;19:358. doi: 10.1186/s12870-019-1954-0 (PMC6697964; doi:10.1186/s12870-019-1954-0)
Supplement: Supplementary file 11 — Table S6. Non-synonymous SNPs validated by High Resolution Melting (HRM) analysis in TU94–2 and Trombay wild accessions of blackgram. (DOCX 17 kb) [file 12870_2019_1954_MOESM11_ESM.docx]

| **Primer code** | **Functional Annotation** | **Codon**  **substitutions** | **Amino acid**  **replacement** | **Types of mutation** | **Alteration in secondary**  **protein structure and prefered ligands as predicted by protein modelling for native and variant form** |
| --- | --- | --- | --- | --- | --- |
| TWSNP 58 | \| \| Cytochrome c-type biogenesis protein CcmE \| \| --- \| \| \| --- \| --- \| | GAT to AAT | Aspartate to Asparagine | Missense | Ligand preference changes |
| TWSNP 63 | [U-box domain-containing protein 13](https://blast.ncbi.nlm.nih.gov/Blast.cgi#alnHdr_1044523950) | GAG to GAT | Glutamate to Aspartate | Missense | Helices number changes |
| TWSNP 68 | [Uncharacterized protein LOC108329961 [Vigna angularis]](https://blast.ncbi.nlm.nih.gov/Blast.cgi#alnHdr_1044567061) | CGA to CAA | Arginine to Glutamine | Missense | Ligand preference changes |
| TWSNP 70 | Cytochrome P450 | AAC to AAA | Asparagine to Lysine | Missense | Ligand preference changes |
| TWSNP 75 | [hypothetical protein LR48_Vigan10g050400 [Vigna angularis]](https://blast.ncbi.nlm.nih.gov/Blast.cgi#alnHdr_920714524) | ATT to GTT | Isoleucine to Valine | Missense | Ligand preference changes |
| TWSNP 88 | [Hypothetical protein VIGAN_01393500 [Vigna angularis var. angularis]](https://blast.ncbi.nlm.nih.gov/Blast.cgi#alnHdr_965659567) | AAG to AAC | Lysine to Asparagine | Missense | Ligand preference changes |
| TWSNP 91 | Probable pre-mRNA-splicing factor ATP-dependent RNA helicase DEAH2 | AAG to AGG | Lysine to Arginine | Missense | Helices number changes |
| TWSNP 93 | [Uncharacterized protein LOC108329963 [Vigna angularis]](https://blast.ncbi.nlm.nih.gov/Blast.cgi#alnHdr_1044567071) | ACC to GCC | Threonine to Alanine | Missense | Both Helices number and  Ligand preference changes |
| TWSNP 98 | Lariat debranching enzyme | AAT to GAT | Asparagine to Aspartate | Missense | Both Helices number and  Ligand preference changes |
| TWSNP 102 | [Probable protein phosphatase 2C 55 isoform X3 [Vigna radiata var. radiata]](https://blast.ncbi.nlm.nih.gov/Blast.cgi#alnHdr_951010971) | AAC to AAA | Valine to Phenylalanine | Missense | No alteration |
| TWSNP 108 | [Mitochondrial fission 1 protein A-like [Vigna angularis]](https://blast.ncbi.nlm.nih.gov/Blast.cgi#alnHdr_1044586322) | GCT to ACT | Alanine to Threonine | Missense | No alteration |
| TWSNP 118 | [DnaJ protein homolog 1 [Vigna radiata var. radiata]](https://blast.ncbi.nlm.nih.gov/Blast.cgi#alnHdr_951036802) | ATG to ATT | Methionine to Isoleucine | Missense | Ligand preference changes |
| TWSNP 124 | [Cell division cycle protein 48 homolog [Vigna radiata var. radiata]](https://blast.ncbi.nlm.nih.gov/Blast.cgi#alnHdr_950963716) | ACC to ATC | Threonine to Isoleucine | Missense | Helices and strands number changes |
