# Supplementary material for: Transcriptome sequencing, de novo assembly, characterisation of wild accession of blackgram (Vigna mungo var. silvestris) as a rich resource for development of molecular markers and validation of SNPs by high resolution melting (HRM) analysis
Source: BMC Plant Biol. 2019 Aug 16;19:358. doi: 10.1186/s12870-019-1954-0 (PMC6697964; doi:10.1186/s12870-019-1954-0)
Supplement: Supplementary file 12 — Table S7. Details of blackgram genotypes used in this study. (DOCX 14 kb) [file 12870_2019_1954_MOESM12_ESM.docx]

| **Serial No:** | **Blackgram accession** | **Pedigree** |
| --- | --- | --- |
| 1 | TU94-2 | TPU-4 x TAU-5 (Mutant of EC-1682000) |
| 2 | Nayagarh | Local selection |
| 3 | Pant-U19 | UPU-1 x UPU-2 |
| 4 | Ku96-3 | PU19 x NP21 |
| 5 | PUSA-3 | L-151 X T9 |
| 6 | PU31 | UPU97-10 x DPU88-31 |
| 7 | DPU88-31 | PLU131 x T-9 |
| 8 | IPU02-43 | DPU88-31 x DUR-1 |
| 9 | IPU94-01 | NP19 x T-9 |
| 10 | IPU07-3 | DPU88-31 x PDU-1 |
| 11 | EC-168200 | Exotic collection from AVRDC, Taiwan |
| 12 | NDU-1 | Sel. 1 x T9 |
| 13 | Pantu-30 | - |
| 14 | KU96-7 | - |
| 15 | Trombay Wild | *Vigna mungo* var. *silvestris* |
| 16 | TAU-1 | T-9 x UM-196 (Mutant of No. 55) |
| 17 | T-9 | Local selection from Bareilly U.P. |
| 18 | LBG-17 | Netiminumu x Chikkuduminumu |
| 19 | EC168058 | - |
| 20 | TU-67 | TAU-1 X KU96-3 |
| 21 | LBG-20 | - |
| 22 | LBG-685 | - |
| 23 | LBG-623 | - |
| 24 | LBG-752 | - |
| 25 | LBG-709 | - |
| 26 | LBG-703 | - |
| 27 | LBG-693 | - |
