# Supplementary material for: Transcriptome sequencing, de novo assembly, characterisation of wild accession of blackgram (Vigna mungo var. silvestris) as a rich resource for development of molecular markers and validation of SNPs by high resolution melting (HRM) analysis
Source: BMC Plant Biol. 2019 Aug 16;19:358. doi: 10.1186/s12870-019-1954-0 (PMC6697964; doi:10.1186/s12870-019-1954-0)
Supplement: Supplementary file 10 [file 12870_2019_1954_MOESM4_ESM.docx]

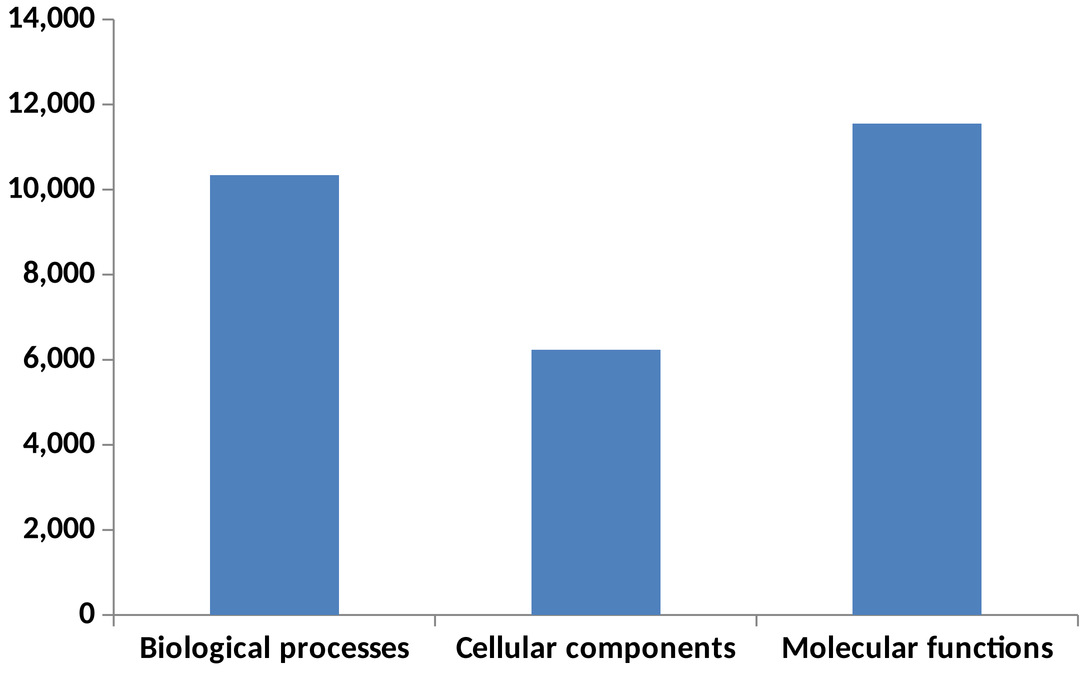
14,000
12,000
10,000
8,000
6,000
4,000
2,000
0
Biological processes
Cellular components
Molecular functions
